# Clinical and Technical Factors Associated With Tunnelled Central Venous Catheter Dysfunction in Haemodialysis: A Multicentre Session‐Level Retrospective Study

**DOI:** 10.1002/nop2.70758

**Published:** 2026-09-09

**Authors:** Verónica Gimeno Hernán, Carla Perez Ingidua, Ana Belén Rivas Paterna, Guillermo Moreno, Maria Jesús Vicente‐Galán, Elena Orgaz‐Rivas, Alfonso Meneses‐Monroy, Ismael Ortuño‐Soriano

**Affiliations:** ^1^ Departamento de Enfermería Facultad de Enfermería, Fisioterapia y Podología, Universidad Complutense de Madrid (UCM) Madrid Spain; ^2^ Servicio de Farmacología Hospital Universitario Clínico San Carlos Madrid Spain; ^3^ Grupo de Investigación Farmacología Clínica Instituto de Investigación Biomédica, Hospital Clínico San Carlos (IdISSC) Madrid Spain; ^4^ Fundación Instituto Español de Investigación Enfermera Madrid Spain; ^5^ Grupo de Investigación Cardiovascular Multidisciplinar Traslacional (GICMT), Área de Investigación Cardiovascular Instituto de Investigación Hospital 12 de Octubre (imas12) Madrid Spain; ^6^ Consulta de Insuficiencia Cardiaca Hospital de Día/Medicina Interna, Hospital Universitario Fundación Alcorcón Madrid Spain; ^7^ Grupo de Investigación en Cuidados Instituto de Investigación Biomédica, Hospital Clínico San Carlos (IdISSC) Madrid Spain

**Keywords:** associated factors, chronic kidney disease, clinical monitoring, haemodialysis, tunnelled central venous catheter, vascular access dysfunction

## Abstract

**Aim:**

To analyse the clinical, technical, and demographic factors associated with tunnelled central venous catheter dysfunction in patients undergoing haemodialysis.

**Design:**

Multicentre, retrospective session‐level observational study.

**Methods:**

We analysed 40,193 haemodialysis sessions from 743 patients with tunnelled central venous catheters. Descriptive and univariate analyses were performed to explore associations between catheter dysfunction and clinical, technical and treatment‐related variables. Dysfunction within a session was defined by a blood flow < 300 mL/min together with abnormal arterial (< −250 mmHg) or venous (> +250 mmHg) pressures at baseline or 60 min.

**Data Source:**

Nefrosoft clinical system (January–December 2021).

**Results:**

As expected from the operational definition, sessions classified as dysfunctional showed lower blood flow and more extreme arterial and venous pressures. Beyond the intradialytic markers included in the operational definition, dysfunctional sessions more frequently involved management‐related measures such as catheter line reversal and the use of 5% heparin locks, as well as catheter placement in the left internal jugular vein and comorbidities such as diabetes and cardiovascular disease. Because line reversal and 5% heparin locks may represent responses to poor catheter performance, these findings should be interpreted cautiously. Sessions with dysfunction also showed lower dialysis efficacy, particularly in male patients. Overall systemic anticoagulant therapy was more frequent in sessions with dysfunction, whereas overall systemic antiplatelet therapy, analysed as a binary variable, was not significantly associated with the event.

**Conclusion:**

Catheter dysfunction is a frequent complication with multifactorial associations. Our findings highlight the importance of systematic clinical and technical monitoring during haemodialysis sessions. Associations identified here are descriptive and hypothesis‐generating; further studies with adjusted analyses are needed before conclusions about prediction or causality can be drawn.

## 1 Introduction

Chronic kidney disease (CKD) is a highly prevalent global condition and a public health concern due to its negative impact on patients' quality of life and the associated healthcare costs. In advanced stages, renal replacement therapy is required, with haemodialysis being the most common option. A functional and durable vascular access is essential for effective treatment. Although arteriovenous fistulas are preferred, they are often unfeasible, leading to the use of tunnelled central venous catheters as either temporary or permanent access devices (Clark et al. 2016).

## 2 Background

Despite their utility, tunnelled central venous catheters are prone to complications, with catheter dysfunction being particularly relevant due to its impact on dialysis efficacy. Dysfunction is typically defined by insufficient blood flow (< 300 mL/min), compromising toxin clearance and reducing dialysis adequacy (Kt/V) (Griffiths et al. 2011; Moya Mejía et al. 2006). Zhao et al. (2022) showed that reduced catheter flow correlates with higher mortality in haemodialysis patients, highlighting the need to preserve vascular access functionality. Inefficient dialysis due to catheter dysfunction can also cause uremic toxin accumulation and metabolic acidosis, increasing hospitalisation risk (Salvador García et al. 2019).

Factors influencing catheter dysfunction include patient characteristics—such as coagulation status, body mass index, and comorbidities like diabetes or hypertension—as well as technical variables like catheter material, anatomical location, and dwell time (Brzosko et al. 2008; Ge et al. 2012). For instance, femoral catheters show higher dysfunction rates than jugular ones due to thrombosis and mechanical compression (Kennard et al. 2017).

The concurrent use of antiplatelet and anticoagulant therapy in patients with CKD may also affect catheter patency. While such therapies are essential for cardiovascular risk management, their influence on catheter functionality remains debated. Some studies suggest that prolonged use may increase bleeding at the tunnel site, promoting intraluminal clot formation and subsequent dysfunction (Forero Villalobos and Murtagh Toro 2019). Wang et al. (2016) assessed anticoagulation strategies and found that specific regimens could reduce obstruction without significantly increasing bleeding risk.

In clinical practice, catheter dysfunction is usually managed reactively, often after complications have occurred, contributing to higher hospitalisation rates and treatment costs (Castro De La Nuez et al. 2019).

## 3 The Study

### 3.1 Aim(s) and Objective

This study aims to analyse and describe the clinical, demographic, and technical factors associated with tunnelled central venous catheter dysfunction in haemodialysis patients, in order to generate evidence that supports prevention and optimised management. Specific objectives are to assess the association between dysfunction and session‐level clinical and technical characteristics, including blood flow and catheter site, and to evaluate its relationship with Kt and dialysis efficacy. Additionally, this study contributes to exploring the potential influence of antiplatelet and anticoagulant use on vascular access patency.

## 4 Methods/Methodology

### 4.1 Design

A retrospective observational study was conducted at the session level in patients with CKD undergoing haemodialysis with tunnelled catheters.

### 4.2 Sampling and Recruitment

A convenience sampling strategy was used based on the total number of haemodialysis sessions available for analysis during the study period. These sessions corresponded to patients with tunnelled central venous catheters undergoing chronic haemodialysis in centres belonging to a national renal foundation in Spain.

### 4.3 Population and Sample

The target population consisted of patients enrolled in haemodialysis programmes using tunnelled central venous catheters in specialised nephrology centres. All haemodialysis sessions recorded between January and December 2021 in the Nefrosoft system across 15 participating centres were included.

### 4.4 Inclusion and/or Exclusion Criteria

Inclusion criteria included adult patients (over 18 years) receiving chronic haemodialysis, who had used a tunnelled central venous catheter for at least 1 month and had complete clinical records available. Exclusion criteria included hospitalised patients in whom exclusive use of the catheter for haemodialysis could not be ensured during the observation period.

### 4.5 Data Sources/Collection

Secondary variables were grouped into *static* and *dynamic* categories based on whether they could change from one session to the next. Static variables comprised sociodemographic characteristics (age, sex), comorbidities, the aetiology of kidney disease, baseline medication and the catheter's insertion site. Dynamic variables included session‐level factors that may vary between or within sessions, such as session duration, urea clearance (Kt), circuit pressures and catheter line configuration. Static variables were extracted from patient records prior to the index session, whereas dynamic variables were captured from the haemodialysis monitor during the treatment.

For reproducibility, key exposure variables were operationalised according to their temporal relationship with the dysfunction event. Catheter line configuration (normal vs. reversal) was recorded as a session‐level technical variable documented during the haemodialysis session. Catheter locking solution was recorded as the intraluminal solution instilled into the catheter at the end of the session and should therefore be interpreted as a session‐level management‐related variable rather than as a baseline exposure. By contrast, systemic antiplatelet therapy and systemic anticoagulant therapy were extracted from the patient's medication record and analysed as baseline treatment variables. These systemic therapies should not be interpreted as equivalent to catheter locking solutions.

#### 4.5.1 Outcome Definition

Tunnelled central venous catheter dysfunction within the same haemodialysis session was defined as a binary variable according to the operational rule implemented in Nefrosoft. The dialysis machine automatically generated a dysfunction flag when the blood‐flow rate (Qb) fell below 300 mL/min and was accompanied by an abnormal pre‐pump arterial pressure (< −250 mmHg) and/or an abnormal return venous pressure (> +250 mmHg) measured at baseline (0 min) or at 60 min. These thresholds correspond to KDOQI recommendations. Nursing staff subsequently verified each flagged event in the Nefrosoft system. Because the haemodynamic measurements that trigger the dysfunction flag form part of the outcome definition, they were not interpreted as independent predictors but as intradialytic markers of dysfunction. Baseline patient characteristics and treatment‐related variables were analysed separately from these intradialytic manifestations.

### 4.6 Data Analysis

A structured database was built in Microsoft Excel to support statistical analysis. Due to the large volume of records, Python programming was used to clean, structure, and process the data efficiently and accurately.

A descriptive statistical analysis was performed to summarise and interpret the variables involved. Frequency distribution was used to describe qualitative variables. For quantitative variables, mean and standard deviation (SD) were applied when the assumption of normal distribution was met. Normality was assessed using the Shapiro–Wilk test. If normality was not assumed, the median and interquartile range were used as appropriate descriptive measures.

To assess the association between clinical, technical, and demographic variables and intradialytic catheter dysfunction within the same session, descriptive and univariate analyses were performed. For qualitative variables, the chi‐square test or Fisher's exact test was used, as appropriate. For quantitative variables, Student's *t*‐test was used for normally distributed variables and the Mann–Whitney U test for non‐parametric variables. For event‐based comparisons between arterial and venous dysfunction events, continuous variables were compared using independent‐samples *t*‐tests, with Welch correction applied when equal variances were not assumed.

Only sessions with complete available information for the variables included in each analysis were considered. Data consistency was reviewed through structured cleaning procedures, and implausible or clearly inconsistent values detected during data processing were checked against the available records whenever possible. Because multiple sessions belonged to the same patient, the lack of adjustment for within‐patient clustering may have inflated statistical significance; therefore, the present findings should be interpreted as descriptive and hypothesis‐generating.

All statistical analyses were performed using SPSS v26 and STATA. A significance level of *p* < 0.05 was considered statistically significant.

### 4.7 Ethical Considerations

Clinical data were accessed following a formally approved and structured procedure, which authorised the use of relevant information stored in the Nefrosoft software implemented across the participating dialysis centres.

The process required institutional authorisation and the signing of confidentiality and data protection agreements. The principal investigator was granted secure access to the Nefrosoft platform via a personalised login and was trained by the nursing leadership team on system navigation and data extraction procedures.

Nefrosoft served as a central tool for the systematic collection of both clinical and technical variables. Data reliability was maintained through anonymization using unique numeric identifiers for each patient and verified through random sampling and consistency checks against original clinical records.

This study was conducted in accordance with internationally recognised ethical standards for research involving human participants, including those outlined in the Declaration of Helsinki and the European Union General Data Protection Regulation (EU Regulation 2016/679). The research protocol was submitted to and approved by the Clinical Research Ethics Committee, which issued a favourable opinion on 11 July 2022. Informed consent was waived by the Ethics Committee due to the retrospective nature of the study and the use of anonymised routinely collected clinical data.

Regarding personal data processing, confidentiality and data protection were ensured throughout the study in accordance with Spain's Organic Law 3/2018 on Personal Data Protection and the Guarantee of Digital Rights. To preserve participant anonymity, strict data coding procedures were implemented, preventing any identification of individuals. Access to the key linking codes to personal identities was restricted solely to the principal investigator, ensuring the ethical and secure handling of sensitive information throughout the research process.

## 5 Results

During the observation period, a total of 743 patients undergoing haemodialysis with tunnelled central venous catheters were evaluated. Of these, 63.7% were female (*n* = 473) and 36.3% male (*n* = 270). The mean age of the cohort was 68.4 years (SD: 14.2), ranging from 20 to 97 years.

Clinically, arterial hypertension was the most frequent comorbidity, present in 84.5% of patients (*n* = 628), followed by diabetes mellitus, with a prevalence of 49.4% (*n* = 367). Cardiovascular disease was identified in 60.1% (*n* = 446), and peripheral arterial disease in 30.6% (*n* = 227). Additionally, 9.2% (*n* = 69) had a history of cerebrovascular disease, and 15.6% (*n* = 95) had an oncological history.

Regarding the aetiology of CKD, diabetic nephropathy was the most common cause (34.3%), followed by vascular (14.4%) and glomerular (12.4%). A significant proportion (24.1%) was classified as unknown aetiology. Only 8.7% had previously received a kidney transplant.

As for technical aspects of treatment, the average duration of haemodialysis sessions was 3.7 h (SD: 0.4). High‐flux haemodialysis was the most commonly used technique (73.7%), followed by post‐dilution haemodiafiltration (25.7%), and pre‐dilution haemodiafiltration (0.5%). The mean body surface area was 1.74 m^2^ (SD: 0.2), and the average height was 163 cm (SD: 14.6).

Concerning vascular access history, 56% of patients had no prior access. Furthermore, 65.4% had never had a native arteriovenous fistula, and 90.3% had never had a prosthetic fistula. Regarding prior tunnelled catheter use, 78.3% had not used tunnelled catheters before, and only a small proportion had experienced multiple episodes.

Most patients were treated with three dialysis sessions per week, consistent with current clinical guidelines for haemodialysis. Regarding adjunct pharmacological treatment, 21% of patients were receiving antiplatelet therapy, while 79.5% were under anticoagulant treatment.

The relationship between clinical, technical, and demographic variables and the occurrence of catheter dysfunction within the same dialysis session is described in Table 1.

**TABLE 1:** Session‐level descriptive analysis according to the presence or absence of intradialytic tunnelled central venous catheter dysfunction (haemodialysis sessions).

	Dysfunction indicator		*p*
	No	Yes	
Mean pre‐pump arterial pressure [mmHg; M(SD)]	−172.2 (40.5)	−215.2 (45.6)	0.000
Mean venous return pressure [mmHg; M(SD)]	142.1 (32.7)	120.9 (49.9)	0.000
Mean transmembrane pressure [mmHg; Me [Q1–Q3]]	30 [8;80]	25.0 [10.3;72]	0.040
Initial blood flow rate [mL/min; M (SD)]	350.7 (51)	257.9 (54.1)	0.001
Initial pre‐pump arterial pressure [mmHg; M(SD)]	−166.9 (50.25)	−207.2 (69.82)	0.000
Initial venous return pressure [mmHg; M (SD)]	139.7 (38.9)	122.01 (56.1)	0.000
Initial transmembrane pressure [mmHg; Me [Q1–Q3]]	30 [7;80]	22 [6;67]	0.000
Blood flow rate at 60 min [mL/min; M (SD)]	358.3 (41)	256 (42)	0.000
Arterial pre‐pump pressure at 60 min [mmHg; M (SD)]	−170.1 (41.2)	−229 (59)	0.000
Venous return pressure at 60 min [mmHg; M (SD)]	142.3 (33.4)	120 (53.5)	0.000
Transmembrane pressure at 60 min [mmHg; Me [Q1–Q3]]	30 [8;80]	25 [10;70]	0.021
Total blood volume processed [litres; M (SD)]	75.6 (17)	56.2 (16.7)	0.001
Catheter lines [*n* (%)]			
Normal	26,160 (98%)	524 (2.0%)	0.001
Reversal	12,631 (96.5%)	454 (3.5%)	
Final Kt [litres; M (SD)]	48.2 (9.6)	39.1 (11.2)	0.000
Haemodialysis duration [hours; M (SD)]	3.6 (0.4)	3.7 (0.5)	0.501
Catheter locking solution [*n* (%)]			
Non locked	374 (85.6%)	63 (14.4%)	0.001
1% Sodium heparin	35,245 (98%)	724 (2%)	
5% Sodium heparin	3060 (95.7%)	137 (4.3%)	
Saline solution	91 (100%)	0 (0.0%)	
Taurolock	107 (100%)	0 (0.0%)	
Urokinase	327 (83.8%)	63 (16.2%)	
Antibiotic	2 (100%)	0 (0.0%)	
Type of haemodialysis [*n* (%)]			
High‐flux HD	29,919 (97.2%)	861 (2.8%)	0.000
Post‐dilution HDF	9011 (98.7%)	121 (1.3%)	
Pre‐dilution HDF	253 (98.4%)	4 (1.6%)	
Ultrafiltration	20 (95.2%)	1 (4.8%)	
Sex [*n* (%)]			
Male	14,873 (97.2%)	428 (2.8%)	0.000
Female	24,333 (97.8%)	559 (2.2%)	
Age [years; M (SD)]	68.8 (14.9)	69.5 (13.5)	0.131
Height [cm; M (SD)]	162.9 (9.8)	162.4 (10.6)	0.062
Body surface area [m^2^; M (SD)]	1.7 (0.2)	1.7 (0.2)	0.630
Previous vascular accesses [number; Me [Q1–Q3]]	1 [0;2]	1 [0;2]	0.000
Previous native arteriovenous fistula [number; Me [Q1–Q3]]	0 [0;1]	0 [0;1]	0.000
Previous tunnelled central venous catheter [number; Me [Q1–Q3]]	0 [0;1]	0 [0;1]	0.000
Previous prosthetic arteriovenous fistulas [*n* (%)]			
0	34,193 (97.8%)	779 (2.2%)	0.000
1	4468 (95.9%)	192 (4.1%)	
2	421 (96.3%)	16 (3.7%)	
3	117 (100.0%)	0 (0.0%)	
Diabetes mellitus [*n* (%)]	20,516 (97.8%)	468 (2.2%)	0.000
Arterial hypertension [*n* (%)]	33,441 (97.5%)	843 (2.5%)	0.920
Cerebrovascular accident [*n* (%)]	7519 (97.2%)	217 (2.8%)	0.031
Cardiopathy [*n* (%)]	24,440 (97.9%)	537 (2.1%)	0.001
Peripheral arterial disease [*n* (%)]	13,592 (97.9%)	293 (2.1%)	0.000
Previous kidney transplants [*n* (%)]	3890 (97.6%)	95 (2.4%)	0.750
SARS‐CoV‐2 [*n* (%)]	5415 (97.1%)	163 (2.9%)	0.000
Tumours [*n* (%)]	5201 (96.6%)	182 (3.4%)	0.000
Tunnelled central venous catheter location [*n* (%)]			
Right femoral vein	163 (93.7%)	11 (6.3%)	0.000
Left femoral vein	987 (100%)	0 (0.0%)	1.000
Right subclavian vein	684 (99.0%)	7 (1.0%)	0.010
Right internal jugular vein	34,331 (99.3%)	231 (0.7%)	0.000
Left internal jugular vein	4637 (94.9%)	250 (5.1%)	0.000
Antiplatelet therapy [*n* (%)]	9224 (97.5%)	235 (2.5%)	0.870
Anticoagulant therapy [*n* (%)]	8847 (97.2%)	253 (2.8%)	0.021

*Note:* Table 1 includes 40,193 haemodialysis sessions (39,206 without and 987 with intradialytic tunnelled central venous catheter dysfunction). Categorical percentages are presented row‐wise using available‐case denominators. Missing data were recorded for haemodialysis modality (*n* = 3), previous prosthetic arteriovenous fistulas (*n* = 7), and catheter line configuration (*n* = 424; 415 without dysfunction and 9 with dysfunction). Catheter locking solution refers to the intraluminal solution instilled at the end of the session and is distinct from systemic anticoagulant therapy.

Abbreviations: HD, haemodialysis; HDF, haemodiafiltration; M (SD), arithmetic mean and standard deviation; Me [Q1–Q3], median and 25th–75th percentiles; *n* (%), frequency and percentage.

Among the 40,193 haemodialysis sessions analysed, 987 sessions (2.5%) met the intradialytic dysfunction criteria. Table 1 presents the session‐level distribution of haemodialysis sessions according to the presence or absence of dysfunction, whereas Table 2 reports dysfunction events according to arterial or venous type. Because a single haemodialysis session may contribute more than one dysfunction event, the counts shown in Table 2 should be interpreted as event‐based data and are not directly comparable to the session counts reported in Table 1.

**TABLE 2:** Event‐based comparison of tunnelled central venous catheter dysfunction events according to arterial or venous type within the same haemodialysis session.

	Type of dysfunction		*p*
	Arterial	Venous	
	*N* = 1346	*N* = 135	
Mean venous return pressure [mmHg; M (SD)]	111 (36.2)	220.4 (57.8)	0.000
Mean pre‐pump arterial pressure [mmHg; M (SD)]	−221.9 (38)	−145.7 (49.4)	0.000
Venous return pressure at 60 min [mmHg; M (SD)]	109.4 (38.8)	226.4 (58.5)	0.000
Arterial pre‐pump pressure at 60 min [mmHg; M (SD)]	−237.8 (49.2)	−144.7 (51.8)	0.000
Initial venous return pressure [mmHg; M (SD)]	110.6 (43.5)	223.1 (69.6)	0.000
Initial pre‐pump arterial pressure [mmHg; M (SD)]	−213.4 (68.2)	−143.3 (57.9)	0.000
Kt final [litres; M (SD)]	39.5 (11.5)	36.5 (8.9)	0.000
Previous vascular accesses [number; M (SD)]	1.3 (1.42)	2 (1.9)	0.000
Total blood processed [litres; M (SD)]	56.3 (13.7)	55.2 (13.4)	0.373
Haemodialysis duration [hours; M (SD)]	3.7 (0.5)	3.8 (0.6)	0.030
Age [years; M (SD)]	70.1 (13.6)	67.1 (13.5)	0.015
Sex [*n* (%)]			
Male	563 (41.8%)	94 (69.6%)	0.000
Female	783 (58.2%)	41 (30.4%)	
Catheter lines [*n* (%)]			
Normal	725 (54.2%)	72 (54.5%)	0.940
Reversed	613 (45.8%)	60 (45.5%)	
Diabetes mellitus [*n* (%)]	646 (48%)	66 (48.9%)	0.840
Arterial hypertension [*n* (%)]	1157 (86%)	95 (70.4%)	0.000
Cerebrovascular accident [*n* (%)]	303 (22.5%)	9 (6.7%)	0.000
Cardiopathy [*n* (%)]	744 (55.3%)	66 (48.9%)	0.151
Peripheral arterial disease [*n* (%)]	396 (29.4%)	68 (50.4%)	0.000
Previous kidney transplants [*n* (%)]	119 (8.8%)	30 (22.2%)	0.000
SARS‐CoV‐2 [*n* (%)]	236 (17.5%)	11 (8.1%)	0.000
Tumours [*n* (%)]	227 (16.9%)	49 (36.3%)	0.000
Antiplatelet therapy [*n* (%)]	326 (24.2%)	26 (19.3%)	0.190
Anticoagulant therapy [*n* (%)]	333 (24.8%)	59 (43.7%)	0.000
Catheter locking solution [*n* (%)]			
None	91 (6.8%)	2 (1.5%)	0.081
1% Sodium heparin	968 (71.9%)	105 (77.8%)	
5% Sodium heparin	210 (15.6%)	17 (12.6%)	
Normal saline	—	—	
Taurolock	76 (5.6%)	11 (8.1%)	
Urokinase	1 (0.1%)	—	
Type of haemodialysis [*n* (%)]			
High‐flux HD	1171 (87%)	122 (90.4%)	0.614
Post‐dilution HDF	168 (12.5%)	13 (9.6%)	
Pre‐dilution HDF	5 (0.4%)	—	
Ultrafiltration	2 (0.1%)	—	

*Note:* Table 2 reports dysfunction events rather than dysfunctional sessions. Arterial events: *n* = 1,346; venous events: *n* = 135. A single haemodialysis session may contribute more than one event if both arterial and venous dysfunction occur. Therefore, Table 2 is not directly comparable to the session‐level counts shown in Table 1. *p*‐values for continuous variables were obtained using independent‐samples *t*‐tests. When Levene's test indicated unequal variances, the Welch correction was applied. Categorical variables were compared using the chi‐square test or Fisher's exact test, as appropriate. For categorical variables, percentages are presented column‐wise within each dysfunction‐event type. When variable‐specific denominators were used because of missing data, percentages were calculated using the number of events with available data for that variable as the denominator. A significance level of *p* < 0.05 was considered statistically significant.

Abbreviations: HD, haemodialysis; HDF, haemodiafiltration; M (SD), arithmetic mean and standard deviation; Me [Q1–Q3], median and 25th–75th percentiles; *n* (%), frequency and percentage.

As expected from the operational definition, sessions with catheter dysfunction exhibited lower blood flow and more extreme arterial and venous line pressures at both the start and 60 min into the session. These haemodynamic parameters should therefore be interpreted as intradialytic manifestations of dysfunction rather than as antecedent independent factors. Beyond these intradialytic markers, dysfunctional sessions more frequently included management‐related measures such as documented catheter line reversal and the use of 5% sodium heparin as a catheter locking solution. In routine clinical practice, both measures may be implemented once poor catheter performance is suspected or confirmed, and 5% sodium heparin may also be used as part of local management protocols. Therefore, these variables should be interpreted as session‐level management responses rather than as antecedent causal exposures. Baseline characteristics more frequently observed in sessions with dysfunction included catheter placement in the left internal jugular vein and comorbidities such as diabetes, cardiopathy, peripheral arterial disease, and a history of stroke or malignancy. Systemic anticoagulant therapy was also more frequent in sessions with dysfunction, whereas overall systemic antiplatelet therapy, analysed as a binary variable, was not significantly associated with the event. Additionally, a stratified analysis was conducted to evaluate the association between the studied variables and the specific type of dysfunction (arterial versus venous). This stratification allowed the identification of distinct patterns related to the anatomical location of the functional event (Table 2).

Venous dysfunction was associated with markedly elevated venous pressures across all time points, whereas arterial dysfunction was linked to more negative arterial pressures, particularly at the beginning of the session and at 60 min. Patients with venous dysfunction also showed lower final Kt values, a greater number of previous vascular accesses, and slightly longer haemodialysis sessions. From a clinical perspective, venous dysfunction was more frequent among male patients and in those with peripheral arterial disease, tumours, previous kidney transplantation, and anticoagulant treatment. Conversely, arterial dysfunction was more frequently observed in patients with arterial hypertension, cerebrovascular disease, and SARS‐CoV‐2 infection. Age was slightly higher among arterial dysfunction events than among venous dysfunction events. There were no significant differences between dysfunction types in terms of diabetes, antiplatelet therapy, catheter line orientation, or dialysis modality.

**FIGURE 1:**
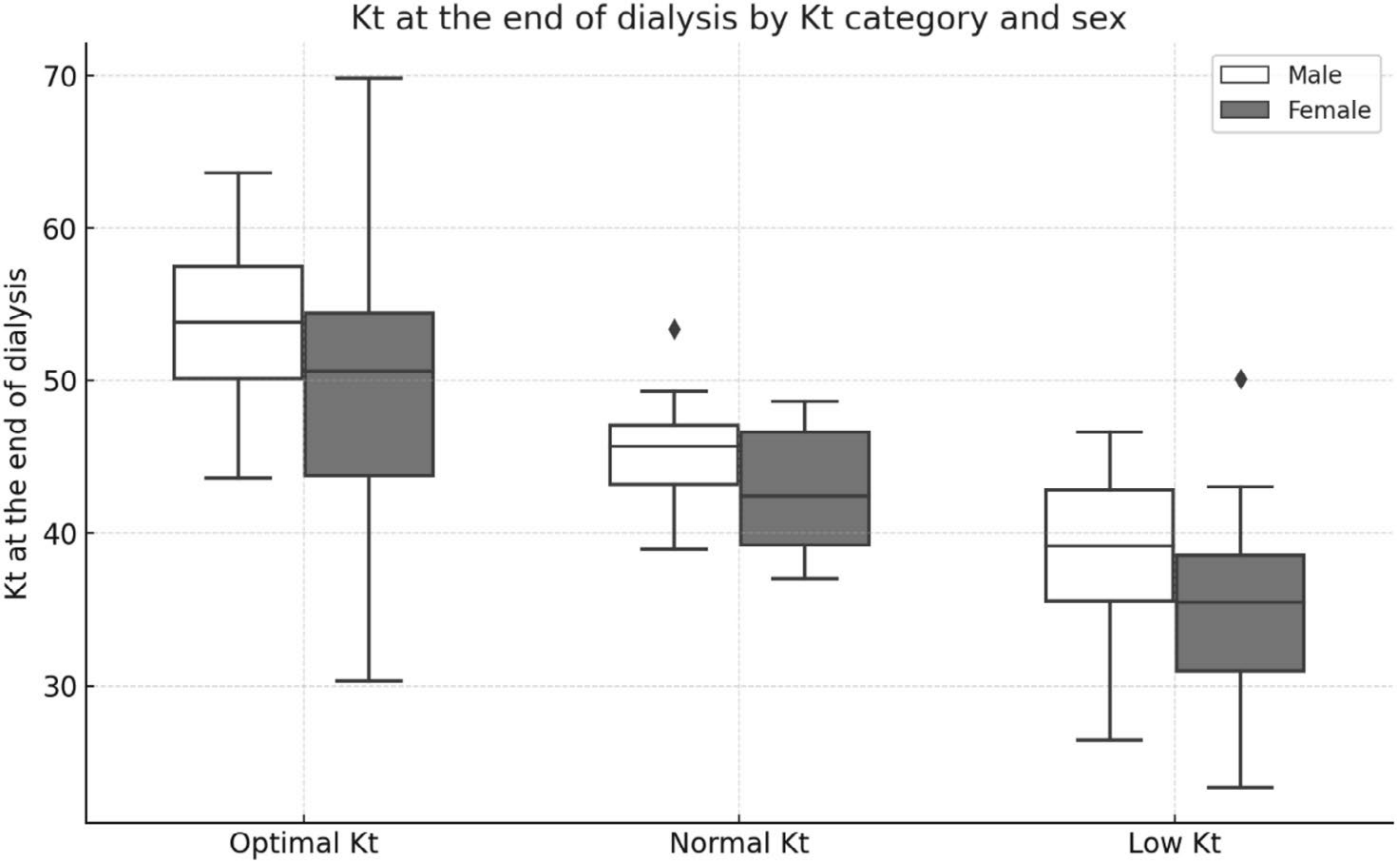
Haemodialysis efficacy category according to final session Kt and patient sex. Final‐session Kt was used as the routinely available marker of dialysis efficacy in the analysed sessions. Categories were defined a priori according to the study variable definition using sex‐specific operational thresholds (women: low < 45  L, normal 40–45 L, optimal > 45 L; men: low < 45 L, normal 45–50 L, optimal > 50 L), as established in the original study framework and used for descriptive stratification of dialysis efficacy. These categories represent literature‐informed operational definitions and should not be interpreted as universal validated adequacy cut‐offs. Because Kt depends partly on body size, comparisons by sex should be interpreted cautiously. (chi‐square test, *p* < 0.001). These Kt categories were used to describe session‐level dialysis efficacy and sex‐related distribution patterns, not to infer causal relationships with catheter dysfunction.

To evaluate the relationship between catheter dysfunction and dialysis efficacy, an analysis was performed using final‐session Kt, which was the dialysis adequacy parameter routinely available across all analysed sessions. Kt categories were defined a priori according to sex‐specific operational thresholds specified in the study variable definition and used consistently in the original study database and thesis framework (women: low < 40 L, normal 40–45 L, optimal > 45 L; men: low < 45 L, normal 45–50 L, optimal > 50 L). These categories were literature‐informed operational definitions used for descriptive stratification of dialysis efficacy and should not be interpreted as universal validated adequacy cut‐offs. Because Kt depends partly on body size, comparisons by sex should be interpreted cautiously. As shown in Figure 1, 24.7% of sessions were classified as low Kt, 20.8% as normal Kt, and 52.6% as optimal Kt. A categorical analysis by sex showed that a higher proportion of low Kt sessions occurred in male patients (41.4%), whereas optimal Kt values predominated among female patients (65.6%), with a statistically significant difference (chi‐square test, *p* < 0.001). These Kt categories were used to describe session‐level dialysis efficacy and sex‐related distribution patterns, not to infer causal relationships with catheter dysfunction.

The analysis of antithrombotic therapy showed that overall antiplatelet use was not significantly associated with dysfunction in the binary analysis presented in Table 1, whereas type‐specific analyses revealed differences between individual agents (Table 3). These findings should be interpreted cautiously, since they may reflect heterogeneity between drugs within the same therapeutic group and possible confounding by indication rather than direct effects on catheter performance. Regarding anticoagulants, enoxaparin and tinzaparin were most frequently associated with dysfunction events, whereas combined regimens such as enoxaparin with warfarin or warfarin with Hemovás showed lower frequencies; however, these findings should also be interpreted in the context of the patients' underlying clinical profile and treatment indication (Table 4).

**TABLE 3:** Influence of systemic antiplatelet therapy type on the occurrence of tunnelled central venous catheter dysfunction within the same haemodialysis session.

Type of antiplatelet	Dysfunction indicator		*p* ^a^
	No TCVC dysfunction	Yes TCVC dysfunction	
Acetylsalicylic acid 100 mg	7655	224	0.000
Acetylsalicylic acid 100 mg/Clopidogrel 75 mg	1628	26	
Acetylsalicylic acid 300 mg	156	0	
Clopidogrel 75 mg	3477	69	
Dipyridamole 100 mg	283	15	
Prasugrel 5 mg	164	18	
Ticlopidine 250 mg	150	0	

*Note:* A significance level of *p* < 0.05 was considered statistically significant.

Abbreviation: CVCT, tunnelled central venous catheter.

^a^ Chi‐square test, grouping variable: dysfunction indicator.

**TABLE 4:** Influence of systemic anticoagulant therapy type on the occurrence of tunnelled central venous catheter dysfunction within the same haemodialysis session.

Type of anticoagulant	Dysfunction indicator		*p* ^a^
	No TCVC dysfunction	Yes TCVC dysfunction	
Enoxaparin	5231	237	0.001
Bemiparin	712	16	
Tinzaparin	128	51	
Enoxaparin/warfarin	1953	14	
Warfarin	5083	78	
Warfarin/hemovás	139	4	

*Note:* A significance level of *p* < 0.05 was considered statistically significant.

Abbreviation: CVCT, tunnelled central venous catheter.

^a^ Fisher's exact test.

## 6 Discussion

The study population was broadly consistent with haemodialysis populations described in Spain and other international settings in terms of age and comorbidity burden, although the predominance of women in our cohort differs from national registry data, where men account for approximately two‐thirds of the haemodialysis population (Castro De La Nuez et al. 2019; Mahillo 2022; Sociedad Española de Nefrología 2022; Otero et al. 2010; Gorostidi et al. 2018; Llisterri et al. 2021; International Society of Nephrology 2023; Fissell et al. 2013). Reports from Fundación Renal and other national units also describe a mean age close to that observed in our study and confirm the frequent use of tunnelled central venous catheters in routine practice (Servicio Madrileño de Salud 2022; Fundación Renal Iñigo Álvarez de Toledo 2021). These differences in sex distribution should be interpreted in the context of the specific clinical profile of patients dialysed through tunnelled catheters.

The causes of CKD and the comorbidity profile observed in our cohort were also broadly in line with previous reports, with diabetic nephropathy as the most frequent aetiology and diabetes mellitus and arterial hypertension as the most prevalent associated conditions (Mahillo 2022; Sociedad Española de Nefrología 2022; Otero et al. 2010; Gorostidi et al. 2018; Llisterri et al. 2021). The higher proportion of diabetic nephropathy in our sample may reflect the specific characteristics of catheter users and should be considered when interpreting the findings (Mahillo 2022). Overall, these data support the representativeness of the cohort while highlighting that tunnelled‐catheter populations may differ in some relevant clinical characteristics from the wider haemodialysis population.

Haemodialysis treatment characteristics were likewise consistent with current practice. The average session duration and weekly treatment frequency were comparable to national reports, and the distribution of dialysis modalities, including the proportion of haemodiafiltration sessions, was similar to that described in registry data and international practice‐pattern reports (Castro De La Nuez et al. 2019; Mahillo 2022; Fundación Renal Iñigo Álvarez de Toledo 2021; Dyalisis Outcome and Practice Patterns Study 2021). Dialysis adequacy is known to depend on factors such as blood flow, dialyser performance, treatment time, and technique (Rocco et al. 2015), and prior studies have shown reduced effectiveness in patients dialysed through catheters compared with arteriovenous fistulas (Canaud et al. 2023). In this context, the use of ionic dialysance‐based Kt measurements in every session provides a practical and reliable approach to monitoring dialysis dose and identifying patients who may be more likely to receive suboptimal dialysis dose, particularly according to sex and body size (Rocco et al. 2015; Maduell et al. 2008).

Regarding catheter placement, the right internal jugular vein was the most commonly used site. This contrasts with findings by Clark et al. (2016), who suggested the left internal jugular vein may yield better performance. Meanwhile, Ge et al. (2012), in a systematic review, concluded that femoral and subclavian catheterisations carry higher thrombotic risk, while internal jugular and femoral sites have fewer mechanical complications—results consistent with Silverstein et al. (2018) and likely explaining the predominance of jugular catheters in our sample.

Regarding catheter‐related management, a key issue in interpreting the present findings is the session‐level, cross‐sectional nature of the analysis. By definition, sessions classified as dysfunctional showed lower blood flow and more extreme arterial and venous line pressures; therefore, these haemodynamic variables should be understood as contemporaneous intradialytic manifestations of dysfunction rather than as independent predisposing factors. The same interpretive caution applies to catheter line reversal and to the use of 5% sodium heparin as a locking solution. In routine haemodialysis care, both measures are often implemented once poor catheter performance has already become evident, and 5% sodium heparin may also form part of local management protocols for suspected dysfunction. Consequently, their association with dysfunction is more plausibly explained as a response to poor catheter function than as evidence of a primary causal role. In this context, reverse causality and confounding by indication are highly relevant considerations (Wang et al. 2016; Natale et al. 2024; Firwana et al. 2011; Neusser et al. 2021; Gónzalez Martínez et al. 2014; Arribas Cobo et al. 2016).

In this study, 1% and 5% sodium heparin were the most commonly used locking solutions and were more frequently documented in sessions with dysfunction. However, this finding should not be interpreted as evidence that these locks caused catheter failure. In a retrospective, cross‐sectional, session‐level analysis such as the present one, locking solutions are recorded as part of routine care delivered within or at the end of the session, and 5% sodium heparin may be selected in response to previous or suspected catheter dysfunction as part of local management protocols. Therefore, the observed association is more appropriately interpreted as reflecting clinical decision‐making in the context of poor catheter performance than as a direct harmful effect of the locking solution itself. This interpretation is also consistent with the broader literature, in which several studies have evaluated different locking strategies without demonstrating a universally superior approach. Wang et al. (2016) suggested a prophylactic benefit of recombinant tissue plasminogen activator, a finding also supported by Firwana et al. (2011). In addition, more recent studies have suggested that taurolidine‐based solutions may reduce catheter dysfunction and improve catheter patency, particularly when combined with citrate (Neusser et al. 2021; Gónzalez Martínez et al. 2014; Arribas Cobo et al. 2016). For this reason, the present findings should be regarded as exploratory and hypothesis‐generating rather than as evidence of a direct causal effect of 5% sodium heparin locks.

A similar consideration applies to pharmacological treatment. In the global binary analysis, antiplatelet therapy was not significantly associated with catheter dysfunction, whereas type‐specific analyses showed differences between individual agents. This apparent discrepancy may reflect heterogeneity within the treatment group and differences in clinical indication, rather than a uniform pharmacological effect on catheter patency. The same reasoning applies to systemic anticoagulant therapy, since its more frequent use in dysfunctional sessions may reflect the underlying vascular risk profile of the patients and the clinical decisions surrounding catheter management, rather than a direct causal relationship with dysfunction. Therefore, these pharmacological findings should be regarded as exploratory and hypothesis‐generating, rather than as evidence of direct protective or harmful effects on tunnelled central venous catheter function (Wang et al. 2016; Natale et al. 2024).

### 6.1 Strength and Limitations of the Work

The results of this study should be interpreted with caution. Some observed associations, particularly those involving higher blood flow, haemodiafiltration, line reversal, or specific lock solutions, may reflect reverse causality or confounding by indication rather than true protective effects, as these factors can represent markers of better catheter performance or rescue measures applied in response to dysfunction. In addition, the statistical analysis was univariate and did not account for the non‐independence of repeated sessions within the same patient, which may have inflated statistical significance and limits the interpretability of the findings to a descriptive, hypothesis‐generating level.

Further limitations should also be acknowledged. This was a retrospective, cross‐sectional session‐level analysis based on routinely collected haemodialysis‐session data; therefore, the dataset was designed to capture variables documented during or around each session rather than catheter‐level physiological mechanisms or insertion‐related procedural details. As a result, the study could not evaluate relevant procedural factors such as insertion technique, operator experience, exact catheter tip design, or detailed catheter tip position, all of which may influence catheter performance. Likewise, fibrin sheath formation—a major physiological determinant of catheter failure—was not systematically assessed in this dataset because it requires imaging or catheter‐focused evaluation beyond the scope of routine session‐level monitoring. For the same reason, the study was not designed to disentangle longitudinal catheter‐level mechanisms, but rather to describe how dysfunction was associated with the variables recorded within each haemodialysis session. In addition, the use of electronic health records may have introduced bias due to missing or inconsistently recorded data. Because all data came from centres within the same institution, generalisability may be limited. Medication use was analysed at session level without accounting for treatment duration, adherence, cumulative dose, or full clinical indication. Finally, outcome misclassification and Type I error due to multiple comparisons cannot be excluded.

#### 6.1.1 Implications for Clinical Practice

Despite these limitations, the findings of this study provide valuable insights for clinical practice in haemodialysis care. The observed associations between catheter dysfunction and sessional technical parameters—such as low blood flow, extreme arterial or venous pressures, and line reversal—support the relevance of careful intradialytic monitoring during routine care. In this context, active surveillance may help clinicians and nursing staff identify early warning signs of poor catheter performance and respond promptly to reduce dialysis inefficacy and vascular access‐related complications.

Similarly, the observed associations involving pharmacological treatment and vascular access history should not be interpreted as evidence of causal risk, but they may help contextualise catheter dysfunction within the broader clinical profile of patients receiving haemodialysis. These findings therefore support a cautious, individualised approach to vascular access assessment and reinforce the value of standardised TCVC care protocols.

Because the present study was descriptive and univariate, its results should be considered hypothesis‐generating. Their main practical implication is to support structured monitoring and clinical vigilance, while future adjusted studies should determine whether any of these variables can contribute to clinically useful risk‐stratification approaches or, if appropriate, to future predictive tools.

### 6.2 Recommendations for Further Research

Given the multifactorial nature of TCVC dysfunction, future research should focus on adjusted analytical approaches that can better disentangle baseline characteristics, intradialytic manifestations, and management responses. If these associations are confirmed in appropriately modelled datasets, subsequent studies could then explore the development and validation of risk‐stratification or predictive tools for clinical use.

Additionally, it would be relevant to explore the role of nursing‐led interventions in the prevention and management of TCVC complications, evaluating their impact on catheter survival and patient‐reported outcomes. Further investigation into sex‐specific responses and anatomical considerations, such as catheter site selection, may reveal personalised approaches to vascular access care.

Finally, multicentre randomised controlled trials should evaluate the comparative effectiveness of anticoagulation strategies and catheter maintenance protocols, including the use of novel sealing agents or locking solutions, in preventing catheter‐related dysfunction and infection.

## 7 Conclusion

Tunnelled central venous catheter dysfunction is a common complication in haemodialysis with a negative impact on treatment efficacy. This study described session‐level clinical, technical, and anatomical variables associated with intradialytic catheter dysfunction, including low blood flow, extreme extracorporeal pressures, line reversal, and left internal jugular placement.

Dialysis efficacy (Kt) was significantly lower in sessions with dysfunction, especially among male patients. Although antithrombotic therapy was common, only some drug‐specific categories were more frequently observed in sessions with dysfunction, and these findings should be interpreted as exploratory because they may reflect management decisions and confounding by indication rather than direct causal effects.

The findings highlight the relevance of continuous clinical and technical monitoring during haemodialysis sessions to identify early signs of catheter dysfunction. However, because the present analysis was descriptive and univariate, these results should be considered hypothesis‐generating and should not be interpreted as evidence of causality or direct predictive capacity. Further studies using adjusted analytical approaches are needed to confirm these associations and to assess their value for prediction.

## Author Contributions

**Verónica Gimeno Hernán:** investigation, data curation, project administration, writing – original draft, writing – review and editing. **Carla Perez Ingidua:** writing – review and editing. **Ana Belén Rivas Paterna:** writing – review and editing. **Guillermo Moreno:** methodology, validation, writing – review and editing. **Maria Jesús Vicente‐Galán:** methodology, validation, writing – review and editing. **Elena Orgaz‐Rivas:** formal analysis, data curation, writing – review and editing. **Alfonso Meneses‐Monroy:** formal analysis, data curation, writing – review and editing. **Ismael Ortuño‐Soriano:** conceptualization, supervision, writing – review and editing. All authors have read and approved the final version of the manuscript and agree to be accountable for all aspects of the work.

## Funding

The authors have nothing to report.

## Ethics Statement

This study was conducted in accordance with internationally recognised ethical standards for research involving human participants, including the Declaration of Helsinki and applicable data protection regulations. The research protocol was approved by the Clinical Research Ethics Committee on 11 July 2022.

## Consent

Informed consent was waived by the Ethics Committee due to the retrospective nature of the study and the use of anonymised routinely collected clinical data.

## Conflicts of Interest

The authors declare no conflicts of interest.

## Data Availability

The data that support the findings of this study are available on reasonable request from the corresponding author. The data are not publicly available due to privacy and ethical restrictions.
